# Development of Electrostatic Microactuators: 5-Year Progress in Modeling, Design, and Applications

**DOI:** 10.3390/mi13081256

**Published:** 2022-08-04

**Authors:** Inga Morkvenaite-Vilkonciene, Vytautas Bucinskas, Jurga Subaciute-Zemaitiene, Ernestas Sutinys, Darius Virzonis, Andrius Dzedzickis

**Affiliations:** 1 Department of Mechatronics, Robotics and Digital Manufacturing, Vilnius Gediminas Technical University, 10257 Vilnius, Lithuania; 2 Laboratory of Electrochemical Energy Conversion, State Research Institute Centre for Physical Sciences and Technology, Sauletekio 3, 10257 Vilnius, Lithuania

**Keywords:** micrometric actuators, electrostatic actuators, conducting polymers

## Abstract

The implementation of electrostatic microactuators is one of the most popular technical solutions in the field of micropositioning due to their versatility and variety of possible operation modes and methods. Nevertheless, such uncertainty in existing possibilities creates the problem of choosing suitable methods. This paper provides an effort to classify electrostatic actuators and create a system in the variety of existing devices. Here is overviewed and classified a wide spectrum of electrostatic actuators developed in the last 5 years, including modeling of different designs, and their application in various devices. The paper provides examples of possible implementations, conclusions, and an extensive list of references.

## 1. Introduction

Recent micro-object manipulation and transportation technologies are mostly based on robotics and manipulation equipment development. Operation with micrometric size objects requires a change in the entire paradigm in manipulation equipment and particularly in the field of related actuators [1]. Microactuators have low energy efficiency compared to the devices of macroscale. The dimensions of these drives are so small that standard kinematic arrangement is not applicable. On the other hand, the small size allows the use of lower mechanical parameters materials due to the dimensional limits of the micromechanisms [2]. With the development of the biological industry, these actuators became a key for living cell positioning, high-speed microscopy, and other micro-scale technologies. New emerging transducer materials such as magnetic nanoparticles [3], metallic nanoparticles [4], expandable micro-spheres, and conductive polymers [5,6] can be used for the design of micro-scale sensors and actuators [7,8]. Despite the used materials, the surface effects and inter-molecular interactions are important in micro-scale; in nanoscale, these phenomena become essential [9].

One of the most common technological solutions used for micropositioning is the implementation of electrostatic actuators. These devices use Coulomb force to create angular or linear displacement of the actuator’s output link. Coulomb force acts on the surface of electrodes and highly depends on the distance between a pair of counter-electrodes and their surface area. Micro/nanoelectromechanical systems (MEMSs/NEMSs) with electrostatic drives have fast response, are power efficient, and fit well for micro- and nanopositioning. Drive operating voltage, response speed, operating stroke, generated force, and the accumulated amount of energy define these actuators’ application area [10]. The electrostatic actuators suffer from the pull-in phenomenon (loss of the equilibrium between attractive and repulsive forces), limiting their performance. This limitation could be overcome by developing actuators based on repulsive force or electrostatic levitation [11].

Electrostatic actuation is the most popular actuation mechanism used in MEMSs/NEMSs due to its many inherent advantages. NEMSs can be integrated into wearable/flexible systems, polymer substrates, or glass devices, also used in manipulation applications, such as nano-surgery, lab-on-chip bimolecular analysis, or nano-orientation in the space environment [12].

Various electrostatic actuators have been developed and utilized in a wide variety of applications, including micromotors [13], microrelays [14,15,16], microresonators [17,18], micromirrors [19,20,21], micropumps [22,23], microvalves [24], and microfilters [25,26,27]. The electrostatic actuators can be classified by operating mode (repulsive/attractive force and electrostatic levitation) [28] and design (parallel plates, comb-drive, scratch-drive, and cantilever-type) [29].

**Figure 1:**
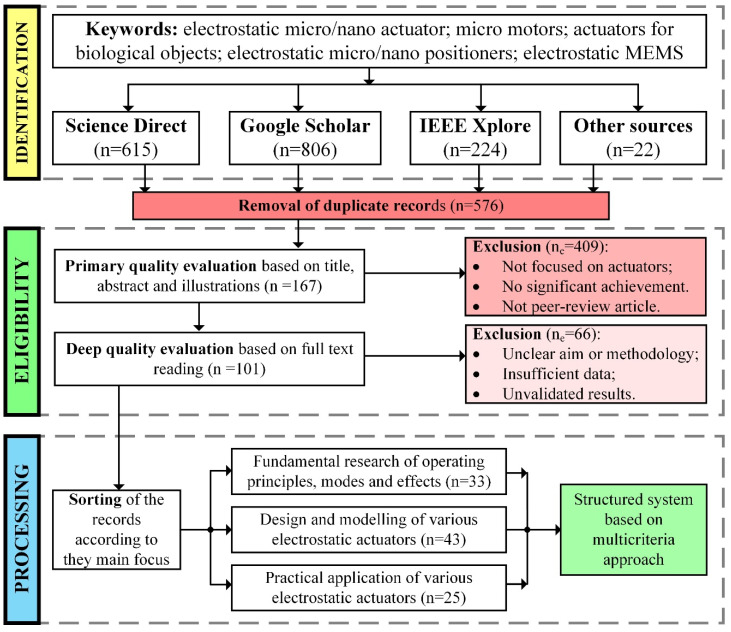
The methodology of review; n—number of articles.

This review covers the field of electrostatic actuators, their operation principle, modeling, and applications. A review of the recent microactuators and manipulation systems covers the period of 2018–2022, mainly including records from three main scientific databases selected according to the provided algorithm (Figure 1).

This review aims to systematize the recent progress in the design research and implementation of electrostatic actuators in order to provide a clear view of the current situation, define most prospective development directions, and formulate guidelines for further research.

## 2. Operating Modes

### 2.1. Attractive vs. Repulsive Force

**Figure 2:**
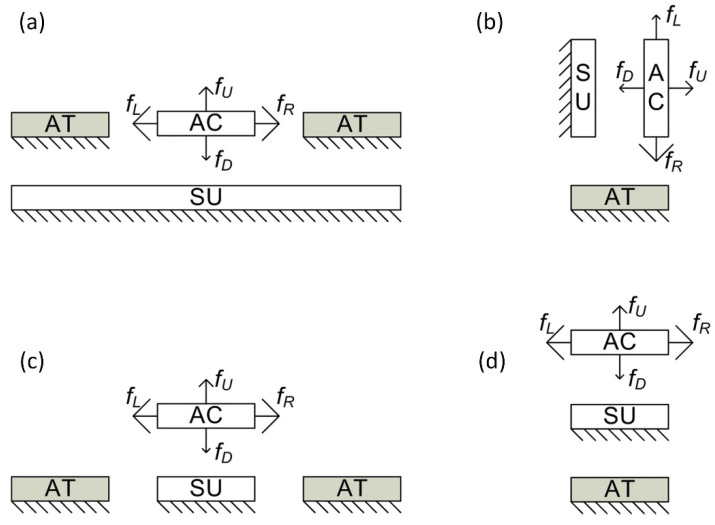
Various repulsive actuators. The AT is the attracting electrode, the SU is the suppressing electrode, and AC is the actuated electrode. (**a**) Levitation of comb-drive actuators. (**b**) In-plane repulsive actuator. The movement direction of AC is to left or right. (**c**) Out-of-plane repulsive actuator. (**d**) Three-layered repulsive actuator. $f_{L}$; $f_{U};\;f_{R}$;$\;f_{D}—$attractive forces. Adapted from [34].

There are two main electrostatic actuator operating modes: attractive mode and repulsive mode (Figure 2). Attractive mode actuators (AFAs) use attractive forces acting between charged electrodes to cause displacement by decreasing the gap between them. One of the biggest disadvantages and factors limiting the development of such actuators is geometric constraints, especially minimal possible gap size between electrodes. When the mechanical restoring force in parallel-plate or comb-type actuators cannot overcome the electrostatic force, the microstructures collapse. At this moment, displacement of the actuator becomes uncontrollable; in addition, this effect can cause damage to the structure [30]. To overcome this problem, several technical solutions have been proposed to prevent mechanical contact between electrodes. These solutions include such approaches as the use of an external electrode [31], the use of a bumper structure [32], or the use of large vertical displacement of the comb-drive actuator [33].

Repulsive mode actuators (RFAs) use electrostatic repulsive force to repeal one electrode from the other; therefore, they are safe from collapse [28]. Moreover, higher control voltages could be used since the voltage increase on the electrodes, in the RFA case, increases the actuator’s natural frequency; in the AFA case, the natural frequency decreases. Therefore, displacement amplitude in RFAs, operating in resonance mode, reaches great values without the risk of jumping into collapse. Repulsive-force actuators (Table 1) solved the pull-in problem in such applications as electrostatic mirrors [30,35] and crawling millirobots [36].

### 2.2. Electrostatic Levitation

**Figure 3:**
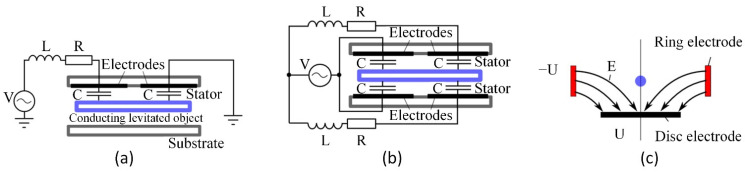
Electric levitation microactuators based on the passive levitation: (**a**) single-stator design, V—electric potential, L—inductance, R—resistance, C—electric charge; (**b**) dual-stator design; (**c**) levitation of a charged particle in a hyperbolic oscillating electric field E (U is the applied electric potential). Adapted from [46].

In the area of microactuators, a wide variety of levitating actuators are known. Typically, they are classified according to the nature of implemented force. The most popular are electric (Table 2) and magnetic levitation microactuators [45]. A comprehensive review of the benefits provided by levitation, operating principles (Figure 3), structures, materials, and fabrication methods are provided in [46]. The key advantage of the levitating microactuators is the elimination of the mechanical attachment between the stationary and moving parts. Such a solution is the way to overcome the system’s dissipative energy and increase the actuator’s velocity due to a vanishing friction influence.

Another problem that can be solved by electrostatic levitation is the pull-in instability of MEMS actuators. Pallay and Towfighian developed the electrostatically levitated beam, which can be used as a switch [47]. The levitation effect was created by applying 100 V voltage pulses on two electrodes placed from the two sides of the beam. In this way, it becomes possible to develop normally closed MEMS switches. In their next paper, the authors showed that this switch could be operated by applying mechanical pressure to a triboelectric generator, becoming a self-powered device [48]; later, they developed the pressure sensing/switching system [49,50,51] and a capacitive MEMS filter that uses electrostatic levitation for actuation and sensing [26]. Other Towfighian group works focus on operating a normally closed MEMS switch, controlled by the electrostatic levitation phenomenon [52]. The MEMS switch combines two mechanisms of gap closing (parallel-plate electrodes) with electrostatic levitation (side electrodes) to provide bidirectional motions [53]. The same group worked on a capacitive MEMS filter that uses electrostatic levitation [25]. This filter can be supplied with very high voltages because it does not suffer from pull-in instability.

**Figure 4:**
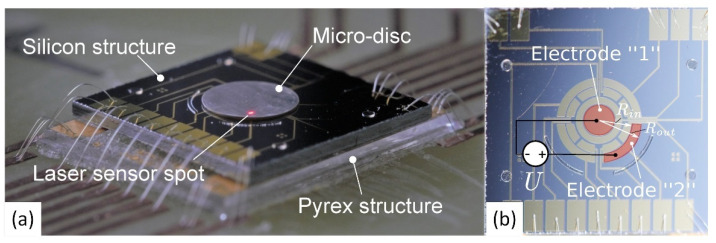
The prototype of microactuator-performed tilting pull-in actuation: (**a**) the fabricated prototype of the microactuator; (**b**) the set of electrodes fabricated on the top of the silicon structure, U—applied electric potential. Adapted from [45].

The electrostatic levitation phenomenon was used in the MEMS microphone to prevent the electrode from pull-in [54,55] and for pressure sensing [51,56,57,58,59]. The concept of electrostatic levitation phenomenon application for the creation of rotating machines is presented in [60]. However, it was not proven experimentally. Electrostatic actuation and inductive levitation were combined in a hybrid levitation microactuator (Figure 4). The actuator consists of the Pyrex structure and the silicon structure with the dimensions: 9.4 mm × 7.4 mm × 1.1 mm [45].

**Table 2:** Electrostatic levitation actuators reported in the last 5 years.

Design	Voltage	Forces/Displacements	Ref.
MEMS switch using electrostatic levitation	100 V	20 µm	[47]
MEMS switch using electrostatic levitation	5.6–150 V	16 µm	[48]
MEMS switch using electrostatic levitation	6–12 V	22 µm	[49]
MEMS microphone using electrostatic levitation	40–100 V	-	[54]
Pull-in-free MEMS microphone	200 V; 16.1 mV/Pa	-	[55,61]
MEMS pressure sensor	Middle voltage 3.5; side voltage 120 V	-	[59]

The non-linear dynamic behavior of a repulsive levitation force actuator was investigated in [62]. Initial impacts cause the appearance of periodic vibration in the output chain due to low friction. Therefore, it is proven that all vibrations are fully periodical.

Nevertheless, the mentioned advantages of levitating microactuators have some serious disadvantages and limitations. The main of them are stability issues and the requirement of accurate control of the gap between the levitating object and surrounding electrodes. To overcome this, closed-loop control systems are typically used. However, such a solution introduces additional complexities and limits possible applications. Another disadvantage of levitating actuators compared to traditional ones is smaller operating stroke, usually not exceeding 50 µm.

## 3. Mathematical Modeling and Main Issues

Modeling of microactuators is essential for analyzing their future properties and can help avoid problems in fabrication, implementation, and control. In electrostatic actuators, the key problem for the modeling (Table 3) is interactions between mechanical and electrostatic parts. A typical way for modeling electrostatic actuators or their systems is the application of the Lagrange II type equation:

(1) $$\left[M\right]\left\{(\ddot{x})\right\}+\left[H\right]\left\{(\dot{x})\right\}+\left[K\right]\left\{x\right\}=\left\{F\left(t\right)\right\}$$

where [*M*], [*H*], [*K*]—matrices of mass, damping, and stiffness, respectively; $\left\{x\right\},\left\{\dot{x}\right\},\;\left\{\ddot{x}\right\}$—vector of the generalized coordinate of the bridge and its derivatives; {*F*(*t*)}—time-dependent load vector, which usually represents the impact of the electrostatic force. In the case of a parallel-plate actuator, electrostatic force can be defined as [63]:

(2) $$F\left(t\right)=\frac{Q{\left(t\right)}^{2}}{A\epsilon }$$

where *Q*(*t*) is the electric charge, *A* is the area of the moving plate, and *ϵ* is the electrical permittivity. Using the relationship between the actuator capacitance charge and voltage of the parallel-plate capacitor, the electrostatic force can also be represented in terms of voltage.

For another type of actuators, the electrostatic force could be obtained from the general rule stating that electrostatic force between two charged conductors is the rate of increase in stored electrical energy with displacement at a constant potential difference between the charged conductors [64]:

(3) $$F_{e}=\frac{1}{2}V^{2}\frac{\partial C}{\partial x}$$

where *F_e_* is the electrostatic force, *C* is mutual capacitance, *V* is applied potential, and *x* is the distance between conductors. In the case of complex shape electrodes, electrostatic force could be obtained using the boundary element approach and splitting electrode surfaces into smaller parts similarly as explained in [65].

Another essential parameter for the development of electrostatic actuators is pull-in voltage. It is voltage defining the moment when electrostatic force overcomes the compensating force of the supporting spring and electrodes stick to each other. This parameter also could be defined using Equations (1)–(3) to develop a lumped parameter mathematical model.

Nevertheless, in a majority of cases, typical models do not fit due to insufficient accuracy or lack of input/output parameters. For example, modeling the dynamics of a parallel-plate electrostatic actuator allowed determining complex temporal interactions between the mechanical and electrostatic non-linearities [66]. The model, which can be applied to any electrostatic actuator, was created in [67]. The authors derived the model with a single input, voltage, or charge to determine pull-in parameters. This model fits for parallel-plate and tilted-plate actuators and evaluates such effects as an external force and large displacement, feedback and parasitic capacitance, and capacitance of the fringing field.

Two-degree-of-freedom (DOF) torsional microactuators with linearly and angularly displaced electrostatic movable plates were analyzed in [68]. The non-linear behavior of the microactuator was analyzed by observing the height and angle dependence on the input voltage.

The pull-in voltage can be calculated by the models based on neural networks; for example, the model of microswitch was designed to determine pull-in voltage using neural networks based on the Levenberg–Marquardt method [69].

**Figure 5:**
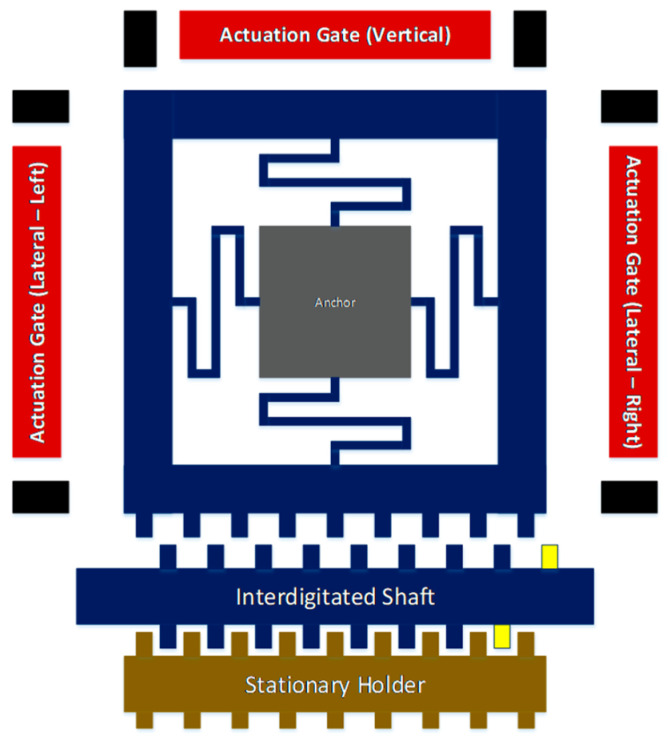
Top view of multiple-degrees-of-freedom electrostatic actuator. Adapted from [70].

A multiple-DOF electrostatic actuator with variable displacement was described in [70]. The authors used an electromechanical model in Matlab/Simulink to investigate the dependencies between the amplitudes of mechanical bouncing and external load. From the simulations, there was noticed an increase in actuator switching time with an increment of increased load on its output chain (Figure 5). As a result of their findings, the authors modified the geometry of the proposed actuator and proposed installing an additional variable capacitor to increase the stability of the actuator start-up [70].

Actuator material properties, such as material grain size, size of the crystals’ ratio to general drive size, and particle surface charge influence pull-in voltage [71]. There is presented comprehensive research on the nanoscale actuator from nanocrystalline silicon (Nc-Si). In this case, specific material properties were assumed according to the Mori–Tanaka micromechanical model. At the same time, the analysis revealed that the dynamical properties of this actuator, such as natural frequency and deflection curve, depend on the actual dimensions, the shape of the drive, and material properties [71].

**Figure 6:**
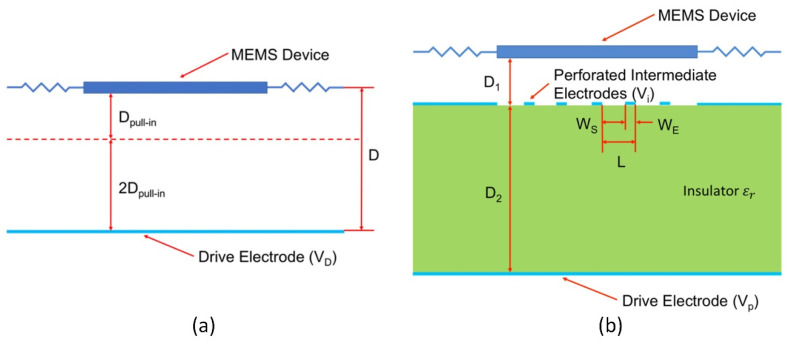
Comparison of electrostatic actuators: (**a**) conventional electrostatic actuator; (**b**) new topology with perforated intermediate electrode, D_pull-in_—controllable stroke, L—electrode pitch, W_s_ —space between electrodes, W_e_—electrode width. Adapted from [73].

Kandula P. et al. introduced an originally developed electrostatic parallel-plate actuator (ESA) with a non-linear active disturbance rejection controller (NADRC). NADRC stabilizes and increases displacement by 99.9% of the ESA full gap [72]. Yu Zhou et al. described the original design of the actuator with three electrodes (Figure 3). This typology aims to decrease drive voltage; therefore, the drive appearance of an intermediate electrode with structural holes and its placement between the electrodes creates the desired drive configuration system [73] (Figure 6). Another way was chosen by Wenguang Wang et al., who developed a mathematical model of the electrostatic actuator, using electric transparent film as an electrode. Using the developed mathematical model, a set of dependencies defining the actuator output forces with respect to the various design of the drives was defined [61]. In the analysis of the synthetic voltage division (SVD) provided by Chong Li et al., this methodology was implemented using dynamic simulation. Technology with an SVD controller ensures stable operation and reduces input voltage. An SVD controller is useful for extra long stroke realization [74]. Cevher Ak et al. presented an artificial bee colony algorithm for calculating the pull-in voltage of microactuators [75].

A. Alneamy et al. presented electrostatic MEMS actuators with contact pads and dimples. Contact pads and dimples minimize electrostatic MEMS actuator dielectric charging, prevent stiction between bottom electrodes and actuators, and eliminate multi-valuedness in actuator response [76].

In nanoscale, electrostatic actuators suffer from surface forces, such as van der Waals and electrostatic forces [77,78,79]. An electrostatically actuated clamp–clamp micro/nanobeam static pull-in voltage closed-loop solution under the action of fringe fields and van der Waals forces was presented in [80]. The stress instability of double-pressed nanoconductors evaluating electrostatic and intermolecular forces was investigated in [78]. The effect of dispersion forces on the dynamic behavior of a micro/nanobeam actuated by electrostatic forces subject to a mechanical shock was evaluated in [79].

The modeling of electrostatic actuators helps to determine interactions between the mechanical and electrostatic non-linearities and pull-in conditions and find the improvements in actuator design to achieve the desired characteristics. By summarizing, it is possible to state that modeling of the electrostatic actuators has two main trends: modeling of the electromechanical systems; modeling of various control methods. Modeling results show that environmental conditions, such as humidity and temperature, have a negative impact on actuators’ performance as well as higher output loads. However, modeling without any experimental evaluation cannot ensure that the model is suitable for the simulation of real devices.

**Table 3:** Modeling of electrostatic actuators reported in last 5 years.

Object	Model	Voltage	Forces/Displacements	Ref.
Mechanical loading and gap influence on the dynamics	Simulink software	60 V	1.3 mN/1 µm	[70]
Electrostatic actuator made of nanocrystalline material	Mori–Tanaka micromechanical model	-	-	[71]
Robust voltage control for an electrostatic microactuator	Non-linear active disturbance rejection controller developed on the electrostatic actuator	1.31–14.32 V	2–4 µm	[72]
Tri-electrode actuator	Topology with perforated intermediate electrode	-	-	[73]
Electrostatic film actuator	Model of electrostatic film actuator using the method of moment (MoM)	500 V	1.125 mN	[61]
Synthetic voltage division	Parallel-plate actuator characteristics with and without series capacitor method	13 V	10 µm	[74]
Fixed–fixed microactuator	Spring mass model, artificial bee colony algorithm	13–56 V	-	[75]
Dimpled electrostatic MEMS actuator	Lumped mass model	45.4 V short actuator, 9.4 V long actuator	-	[76]

## 4. Design

### 4.1. Parallel-Plate Drives

**Figure 7:**
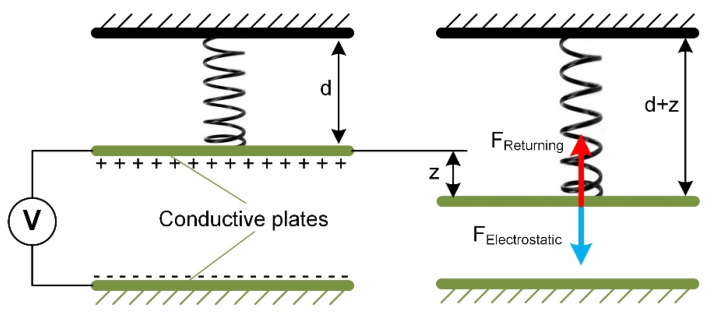
Operating principle of parallel-plate drive, d—initial gap among electrodes, z—displacement of the movable plate. Adapted from [1].

One of the most popular and well-known designs of electrostatic actuators is parallel-plate drives. They are suitable for accurate and controllable actions and fit applications such as microsurgery, microelectronics, and radio frequency switches [66]. The parallel-plate actuator is similar to the typical capacitor, consisting of two separated (by an insulator) parallel plates as electrodes and accumulating static charge (Figure 7). The movable top plate is attached to elastic support, generating the force for return stroke by the vertical z-axis; the fixed lower plate remains unmovable with respect to the ground [81].

The distance between two plates is an essential parameter that determines the request for the maximum actuation voltage. Parallel-plate actuators have an advantage since they have a low operating voltage. The parallel-plate microactuator design can be adapted according to the individual task; for example, they can be used in systems requiring tightly packed micromotors [81].

The DC power supply is needed for electrostatic actuators. One of the interesting solutions for power supply is to use a device-integrated electret [82]. Such a solution allows the development of a low-voltage electrostatic actuator suitable for acoustic actuation.

**Figure 8:**
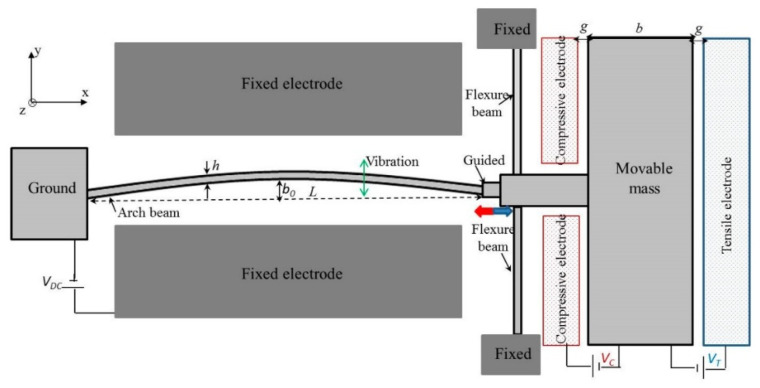
Schematic of the resonators with electrostatic actuation, h—beam thickness, L—beam length, g—gap size, b—width of movable mass. Adapted from [83].

Another interesting application of parallel-plate drives is mechanical resonators [83], with a clamped–guided arch microbeam (Figure 8). Mass is moved bidirectionally in the x-axis due to the deformation of the arch beam. Such a kinematic approach requires a precise definition of the main parameters (thickness and stiffness of the beams, lengths of the beams, and initial curvature) achievable only using powerful modeling and simulation features.

**Figure 9:**
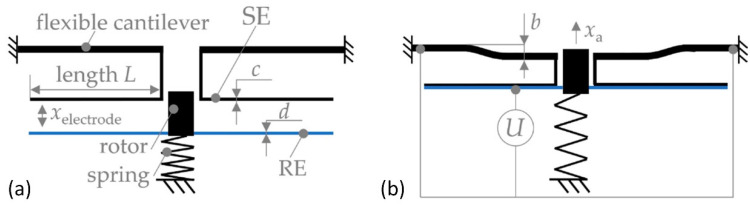
Bending plate actuator: (**a**) initial configuration of the actuator, (**b**) completely pulled-in bending plate actuator, x_a_—maximum displacement of the actuator. Adapted from [84].

Another type of bending plate actuator, designed by Schmitt and Hoffmann, generates a linear stroke of about 20 µm using an electrostatic actuator with a flexible bending plate [84]. This design is based on a rotating scheme of electrodes containing rotor (REs) and stator electrodes (SEs), respectively. The gap between these electrodes is marked as x_electrode_ (Figure 9a). A movable electrode (RE) is attached to the drive rotor, which is attached to the base through the spring. The stator electrode (SE) is fixed to the flexible structural cantilever. When a voltage is supplied, the tips of the electrodes attract each other by overcoming spring stiffness. In case the voltage ceases, the spring will return the actuator to the initial position. Such structure reduces the pull-in voltage of the actuator due to the cantilever’s flexibility and its stiffness variation depending on the distance between the electrodes. Therefore, the bending cantilever is a compromise between a reduction in the pull-in voltage and the maximum displacement.

A summary of the parameters of parallel-plate actuators reported in the last 5 years is provided in Table 4.

The main disadvantage and limitation of parallel-plate drives is their relatively small actuation stroke and output force dependency on the gap size between electrodes. In the case of attractive operating mode, the maximum output force increases while minimizing the gap between electrodes. Nevertheless, the minimal gap size is limited by the collapse effect. In the case of the repulsive mode, actuators are saved from collapse, but their generated output forces decrease by increasing the gap between electrodes.

### 4.2. Comb-Drive Actuators

**Figure 10:**
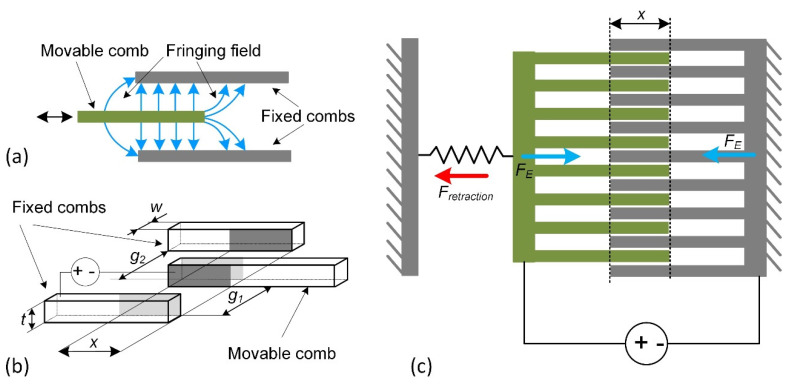
Comb-drive actuator: (**a**) operating principle, (**b**) geometry parameters, (**c**) acting forces. w—width, t—thickness, g1, g2—gaps between combs, x—overlapping length.

Comb-drive actuator design has movable and stationary structures, split in the fingers with the attached combs. Stationary parts are connected to a ground-fixed suspension (Figure 10). Coulomb forces deflect the moving comb structure and generate output displacement. Comb-drive electrostatic actuators can have a lateral and vertical design, which relates to the layout of the comb. Voltage-controlled comb-drive actuators are quite attractive in micro-positioning because of the lateral exposed electrostatic force independent of the position, contrary to the parallel-plate drives.

The biggest disadvantages of comb-drive actuators are moderate driving voltage, the small offset in direct current driving mode, and the large layout area [33]. Nevertheless, these disadvantages can be compensated by a higher displacement rate than other actuator types. A vertical comb-drive actuator is used to achieve greater angular movement. The design of a vertical actuator with a comb drive brings a higher deflection angle in low-voltage cases.

**Figure 11:**
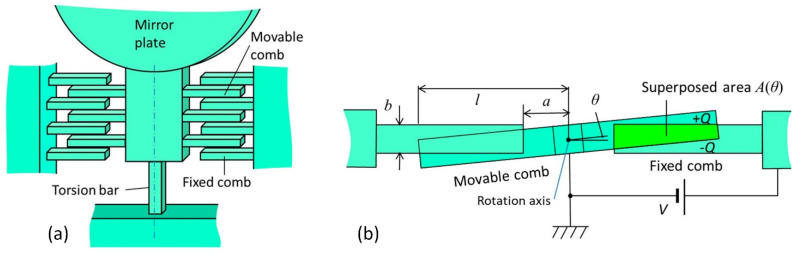
Schematic diagram of torsion bar with electrostatic spring consisting of comb electrodes: (**a**) side view; (**b**) top view; b—thickness; l—the distance between the rotation axis and the end of movable comb finger; a—the distance from the rotation axis to the end of fixed comb finger; θ—rotation angle. Adapted from [89].

Comb-drive actuators are often utilized as resonators, electromechanical filters, and microscrapers [88]. One example of comb-drive actuators is shown in Figure 11. Silicon torsion bars support the micromirror and, at the same moment, work as a returning rotational spring [89].

**Figure 12:**
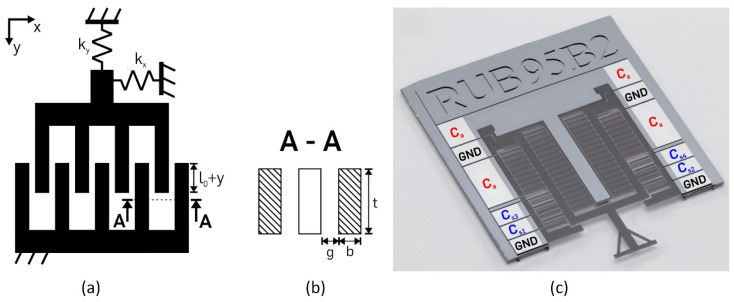
A single-sided electrostatic comb-drive actuator. (**a**) Schematic view, l_0_—finger overlap, y—displacement in y direction, k_x_, k_y_—spring stiffness in x and y directions; (**b**) a cross-section of the finger electrodes with finger gap g, width b, and depth t; (**c**) final processed constant-force microchip with electronic connections. Adapted from [90].

An electrostatic comb drive used in a moveable microsystem with non-linear springs [90] is presented in Figure 12. The device is dedicated to the manipulation of tissue samples and, simultaneously with the motion, can measure the displacement of the probe tip.

The variety of comb drives is typically distinguished by the different geometric parameters and various designs of output kinematic chains. The summary of main parameter combinations reported in the last 5 years is provided in Table 5.

In the field of micro- and nanoactuators, comb drives are preferred rather than parallel drives due to their controllability and other characteristics. Comb structure allows increasing the surface area of the electrodes required to ensure sufficient output force. On the other hand, a comb drive requires more precise machining technologies, and the collapse effect still limits them.

### 4.3. Scratch-Drive Actuator

**Figure 13:**
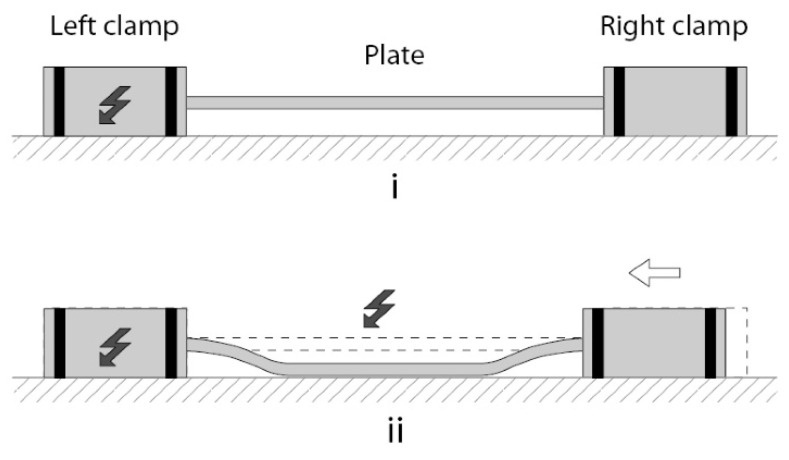
Scratch-drive actuator inchworm-like walking sequence: (**i**) Only the left clamp is activated; (**ii**) a voltage is applied to the plate and causes its inflection. Adapted from [96].

The scratch-drive actuator (SDA) was invented by Akiyama and Shono [94]. In this actuator, the structure is placed on the plate containing two bushings: moving and fixed (Figure 13). By applying voltage, the movable plate is pulled down to the substrate surface, and it creates a displacement of the unpowered part. When the voltage is removed, the unpowered drive returns back to the initial position.

Scratch-drive actuators fit MEMS technology and are especially useful for microoptoelectromechanical systems because they produce linear motion directly, have a potentially large travel range with a relatively large force, control the position and step size very well, and can also operate over a wide speed range [95].

MEMS actuators need higher voltages to generate large displacements; therefore, energy efficiency in the MEMS drives has lower values. Devices with electrostatic scratch actuators require less energy for higher force actuation but require power supplied by RF [97] or substrate [98] sources.

A full-gap tracking system was designed for parallel-plate electrostatic actuators [99]. The triboelectric generator was used for the operation of an electrostatic levitation self-powered normally closed MEMS switch [48]. The switch was opened by applying force to the generator.

Compared to comb- and parallel-plate drives, scratch actuators have a huge advantage—they do not suffer from the collapse effect. On the contrary, they use mechanical friction as a key effect for their operation. However, they have disadvantages too; the main of them is the requirement for a complex voltage pulse generator required for the precise control of the displacement or output force.

### 4.4. Rotary Actuators

**Figure 14:**
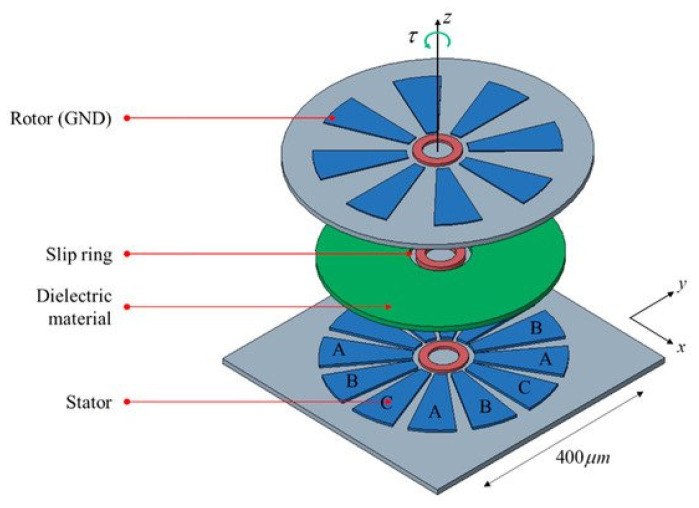
Top-drive electrostatic rotary actuator with a ring-shaped bearing. Adapted from [100].

In general, electrostatic rotary MEMS actuators have a simple design and are known for their high versatility, fast dynamic response, and low power consumption [12,100]. They can be divided into top-drive, side-drive, and wobble-drive actuators [100]. The highest rotor stability is achieved using the side-drive method; however, its torque is low. Large torque can be observed using the top-drive method, but then the stability of the rotor will be lower. Woo et al. proposed a three-phase 12-pole top-drive electrostatic rotary actuator with a ring-shaped bearing (Figure 14). It consists of stator and rotor electrodes, dielectric material, and a slip ring (bearing). The dielectric material helps to isolate the stator from the rotor and increases the capacitance.

The rotation movement of the microdrive simplifies the kinematic scheme in many applications, such as in the grippers, scrappers, or positioners. Rotary movement provides a possibility to improve movement resolution by changing object distance from the rotation axis and eliminating unnecessary gaps on sliders, which can provoke adhesion of some liquids or biological objects during live cell tests. On the other hand, distanced objects from the axis of rotation develop high torques; therefore, real drive size and power can overcome sliding mode ones. The limited range of angular movement is also a limitation of this drive design.

The summary of electrostatic actuators reported in the last 5 years is provided in Table 6.

By summarizing the reported progress in developing the new design of electrostatic actuators in recent 5 years, it can be stated that the designs are the same. The main types are the parallel plate (sliding or bending), comb drive, scratch linear actuators, and their rotational versions. As is seen in Table 4, the main focus of research lies in the modification and optimization of electrode geometry and their allocation to minimize the actuator and, at the same time, increase their operating force or stroke as well as other mechanical characteristics.

## 5. Applications

The potential application of radiofrequency microelectromechanical (RF MEMS) switches can be used in various sensors, amplifiers, resonators, and phase converters [102]. Another prospective application of electrostatic actuators was presented in the work of Chen et al.: a bionic flapping-wing vehicle driven by a mechanical transmission structure and electrical motor [103]. The combination of elastomer and electrostatic actuators has been applied in the tactile display, which is characterized by simple production, compactness, and the ability to work at a wide range of frequencies (0.1–300 Hz) [104]. In the near future, such a device has the possibility to be applied in virtual-reality applications.

**Figure 15:**
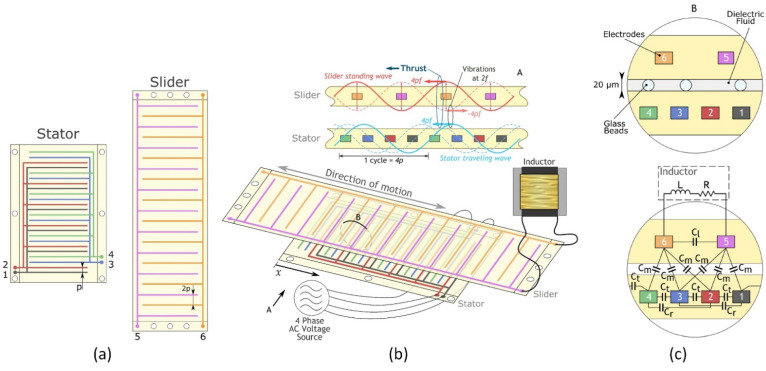
Schematic images of the 2–4-phase resonant electrostatic induction motor: (**a**) the general layout of the films, p—pitch for the stator electrode, 2p—pitch for the slider electrode; (**b**) their typical arrangement, external circuit components, and working principle; and (**c**) a more detailed section view with identification of the discrete capacitive elements. Adapted from [105].

A resonant electrostatic induction micromotor with two to four phases was reported in [105]. It consists of two polymeric films with evenly spaced parallel copper electrodes. The slider film contains two phases, while the stator comprises four phases, as is shown in Figure 15. Due to the different number of phases, the slider and stator have a different pitch between electrodes but the same length of electrode cycles. Consequently, it creates interacting voltage waves generating the tangential electrostatic force between stator and slider. In such a configuration, the motor output force is proportional to the product of stator and slider voltages.

**Figure 16:**
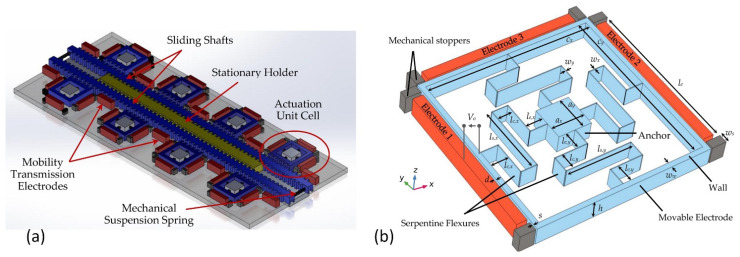
Inchworm motor: (**a**) concept; (**b**) the proposed AUC for the cooperative microactuator system, h—unit cell height, w_w_—width of the cell wall, s—stroke, V_0_—actuation voltage, w_s_—stopper width, l_e_—stationary electrodes length, l_s,y_—spam beam length of y-axis spring, l_e,y_—extension beam length of y-axis spring, l_c,y_—connector beam length of y-axis spring, l_e,x_—extension beam length of x-axis spring, l_c,x_—connector beam length of x-axis spring. Adapted from [106].

Albukhari and Mescheder presented an actuation unit cell (AUC) [106]. The authors created a device capable of large actuation displacements (tens of mm) and forces (tens of N). Such unit cells could be suitable for bone distraction in osteogenesis cases to use as implantable actuators. The design of the system is shown in Figure 16. Pairs of AUCs are implemented in inchworm motors at both sides of two connected sliding shafts (Figure 12a). This device can be used in a cooperative microactuator system (Figure 16b). A summary of the implementation of the electrostatic actuator in the micromotors is provided in Table 7.

**Figure 17:**
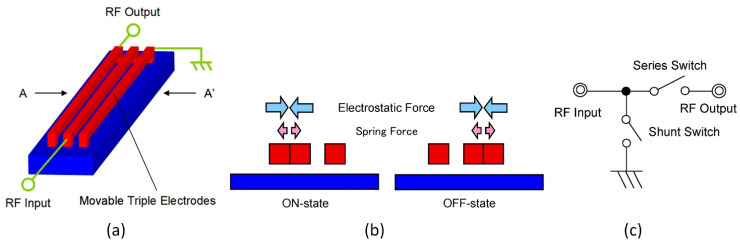
RF-MEMS switch with side movable triple electrodes actuator. (**a**) Overview; (**b**) cross-sectional view between A to A’; (**c**) an equivalent circuit [113].

Figure 17 shows a schematic of the switch (a) and a cross-sectional view (b) [99]. The electrodes come into contact at a switching voltage between the left and center electrodes. The radio frequency (RF) signal goes from the input port to the output port. The center and right electrodes come into contact, and the RF signal goes to the ground in the off state [113]. Part (c) of the figure shows an equivalent circuit. A central electrode is connected to the RF input port, and a left electrode is connected to the RF output port. The shunt switch consists of a central electrode, a right electrode connected to the ground, and an RF input port. Despite the gap between the electrodes of the serial switch, the connection to the ground provides high insulation to reverse in the off state. At a low actuation voltage, the gap makes the response time shorter. This actuator is efficient at an inrush voltage of less than 10 V and responds faster than 10 µs.

Another MEMS switch based on an aluminum beam suspended by the torsion springs over the electrodes was presented in [114]. This switch has a low (4.9 V) pull-in voltage, but contrary to the typical switches, it is equipped with an active opening mechanism allowing recovery in a case of stiction.

**Figure 18:**
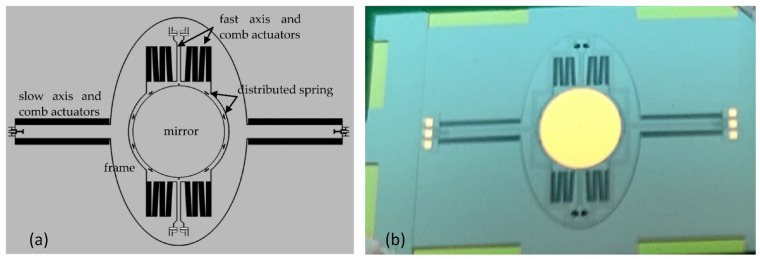
Scheme of the scanner: (**a**) layout of the scanner; (**b**) picture of the prototype. Adapted from [115].

The scanner based on comb actuators with a gimbaled frame as well as an outer (slow) and inner (fast) non-resonant and resonant axes is shown in Figure 18. A distributed spring is used to reduce dynamic deformation. The mirror diaphragm is 4 mm larger than most electrostatic devices, which limits the improvement of other parameters.

**Figure 19:**
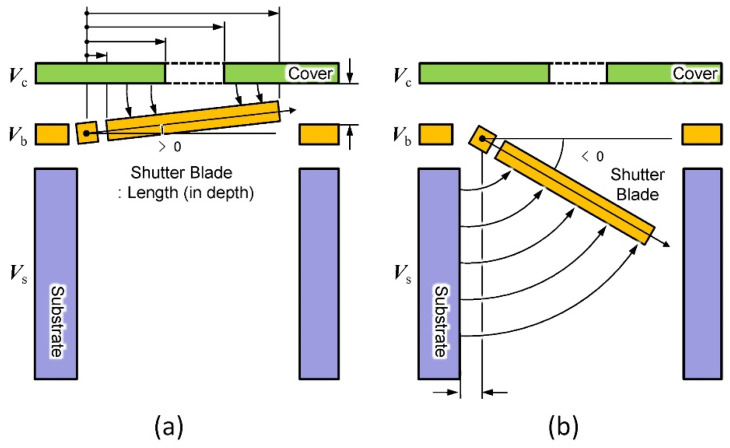
Side view of the three-port electrostatic mechanism. (**a**) The shutter blade locked upward for closing the aperture; (**b**) for opening the aperture, the shutter blade is retracted inward. Adapted from [116].

Figure 19 shows the electrostatic principle of the shutter blade. In part (a) of the figure, the attachment of the shutter blade to the cover plate at a positive angle to the horizontal axis is depicted. Another analytical model is shown in part (b) of the figure when the torsion blade is retracted into the hole.

**Figure 20:**
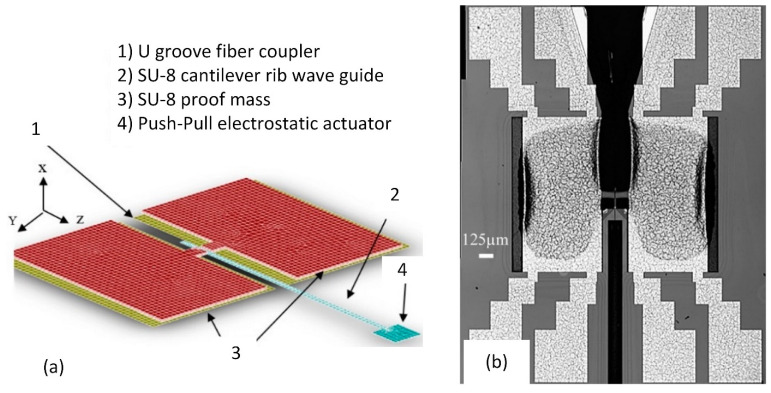
Electrostatic MEMS scanner: (**a**) scheme; (**b**) prototype. Adapted from [117].

In the new model scanner system, the MEMS-based pull-and-push drive is fully integrated with the micro-made waveguide, which increases the robustness (Figure 20). The device is designed to work in tight spaces, as the integrated actuator reduces rigid length as well as the integrated light source and the scanner probe into one package. The MEMS-based push–pull actuator provides a better noise-to-signal ratio and relatively less power consumption during operation compared to commercially available actuators. The push-and-pull actuator mechanism can provide 2D scanning motion via 1D actuation.

Flexible hydraulically reinforced electrostatic actuators less than mm thick were described in [118]. Such an actuator consists of a liquid-filled cavity, the shell of which is made from an elastomer and a metalized polyester. Applying voltage to the electrodes creates a zipping motion by pushing the fluid into the center of tension and forming a raised bump. This type of actuator is able to move both out off-plane and in-plane.

The application area of electrostatic actuators is quite big. RF-MEMS actuators have the largest application area; they are used in satellite vehicles, sensors, amplifiers, resonators, and phase converters. Inchworm motors and electrostatic induction micromotors designed by the electrostatic actuation principle are good candidates for moving microparticles. The fast-growing technology of microrobots requires new small high-performance actuators with low actuation voltage. Electrostatic microactuators can fill this gap successfully.

## 6. Conclusions

Electrostatic actuators make up a big part of the market for micrometric- and nanometric-size operations. They compete with other technologies but have a permanent area of applications. Analysis of existing microdrives developed interesting design variations, their applications, and driver facilities. Obtained properties of recent electrostatic drives show a wide distribution of parameters. For example, the operating voltage varies from 46 V DC (33 V high-frequency pulse) to 800 V DC, which characterizes distinctive power supply systems, control possibilities, and final application limitations. The stroke of the actuators depends on their operating voltage; various designs operate in the range from 8.75 µm to 2.7 mm. This range is very significant, and therefore, the design of drives, fixing method, and control of drive parameters lie apart. Generated forces differ much less, from 0.05 to 1.0 mN.

The application area of electrostatic drives is quite big in micrometric technologies; therefore, the paper shows the prospects of these drives. Recent applications, such as radiofrequency switches, wave propagating environment control, and electrostatic micromotors, do not limit the application possibilities. There are great chances to use electrostatic drives in micromanipulation applications, positioning, and orientation tasks, propagating tools, and sensor tips in the electronic industry and biological applications.

Research reports on electrostatic drives are mostly dedicated to simulation techniques, methods, and technologies. Technological issues for experimental analysis cause such interest in simulations, but they can reveal various physical effects in the drives. Such analysis brings a high value for future implementation, but the accounting of all effects in simulations brings human-dependant evaluation and simplification of real objects, sometimes omitting essential effects in the drive characteristics.

Analysis of recent progress of the micrometric-size electrostatic actuators revealed a direction of improvement as quantitative rather than implementing a new design, technologies, or new physical effects of the operation. Development of the drives went to using better materials, implementing advanced geometrical shapes, and applying higher technological matters. Recent progress in the analyzed area did not make any barrier-breaking news; such a situation continues for a longer period, but advantages in microdrive properties are noticeable, and they will accumulate technological improvement for further quality jump shortly.

## Figures and Tables

**Table 1:** Summary of repulsive-force actuators reported in last 5 years.

Design	Voltage	Forces/Displacements	Ref.
Thin-film repulsive-force electrostatic actuator	500–1000 V	9.03 mN/242–511 µm	[28]
MEMS mirror driven by the repulsive-force actuator	95 V	±43 µm	[30]
Thin-film repulsive-force electrostatic actuator for a crawling millirobot	0–1.2 kV	156 Pa;	[36]
An electrostatic microbeam repulsive-force actuator	200 V	2–15 µm	[37]
An opto-electrostatic repulsive combined actuator for a microgripper	0–400 V	0–750 µm	[38]
MEMS accelerometer using repulsive electrostatic force	0–100 V	0–13 µm	[39]
MEMS micromirror with repulsive electrostatic force	150 V	5 µm	[20]
Repulsive MEMS actuator with displacement sensing capability	10–50 V	0.025–1.8 µm	[40]
MEMS mass sensor with repulsive electrostatic actuation	110 V	0.3–3.8 µm	[41]
Large-displacement vertical electrostatic microactuator	60 V	480 ± 30 µm	[42]
High-energy density arrays of electrostatic actuators	90 V	0.3 mN/230 µm	[43]
Self-excited electrostatic actuators	1.3 kV	±500 µm	[44]

**Table 4:** Design of parallel-plate actuators reported in last 5 years.

Displacement, µm	Force/Voltage	Width/Thickness/Length of the Beam Electrode, µm	Gaps between Electrodes, µm	Ref.
0.7	30 V	2448/1.5/2448 (hexagon)	4.53	[85]
2.4 (stable)	13 V	45/5/2000	5	[66]
-	8.2 V (pull-in)	5/5/125 (beam) 60/1.5/30 (plate)	2.75	[86]
3.5	7.69 (pull-in)	60/-/290 (SSPP)	4.19 and 3.3	[87]
3.5	4.5 (pull-in)	60/-/290 (DSPP)	3.95 and 3.98	

SSPP—single-sided parallel plate; DSPP—double-sided parallel plate.

**Table 5:** Design of comb-drive actuators reported in last 5 years.

Displacement, µm	Force/Voltage	Width/ Thickness/Length of the Finger, µm	Gaps between Combs, µm	Overlapping Length, µm	Ref.
-	12.42 V	3/7/480	-	-	[91]
4.88 degree	24 V	/0.5/	0.6 or 1.8	-	[12]
-	23.4 V	2/7/-	2	-	[92]
±8 degrees	-/120 V	5/20/205	5	190	[89]
40	79 ± 2 µN/25 V	4/20/200	4	100	[90]
-	800 V	2/2/185.3	2	20	[91]
scanning angle 44.3 degrees	30 V	4/-/-	-	no overlapping	[93]

**Table 6:** Electrostatic actuators reported in the last 6 years.

Design	Material	Voltage	Forces/ Displacements	Ref.
MEMS-based parallel-plate microactuator	SU-8	128 VDC	9.89 µm	[81]
Thin-film repulsive-force actuator	copper foil on a polyimide	1000 VAC/43 Hz	9.03 mN 511 µm	[28]
Hexagonal parallel-plate actuator	polysilicon	30 VDC	0.8 µm	[85]
Repulsive-force MEMS electrostatic mirror	-	40 VDC 1 VAC/1.2 kHz	43 µm	[30]
Micro-machined cellular arrays of electrostatic actuators	silicon polysilicon	90 VDC	0.3 mN 230 µm	[43]
Bending plate actuator	silicon-on-insulator (SOI) wafers	54 VDC	230.7 ± 0.9 µm	[84]
Bidirectional, thin-film electrostatic actuator		0–1.2 kVDC	-	[36]
MEMS-based parallel-plate microactuators	SU-8	128 VDC	33 µm	[81]
Multilayer microhydraulic actuators	polyimide layers separated with liquid	50 VDC	-	[101]

**Table 7:** Electrostatic actuators in micromotors reported in the last 5 years.

Design	Voltage	Forces/Displacements	Ref.
Arrayed cellular actuator	46 V	80 mN 678 µm	[107]
Parallel-plate MEMS electrostatic microactuator	95–128 VDC	8.75–9.89 µm	[81]
Three-phase electrostatic actuation mechanism	-	-	[108]
Electrostatic inchworm motor	60 V	250 µm, 0.05 mN	[109]
Side-drive electrostatic micromotor			[110]
Electromagnetically levitating hybrid microactuator	27–33 V, 10 MHz	34–45 µm	[45]
Electrostatic actuator in robot	500 V	2.7 mm	[111]
	800 V	2.1 mm	
	700 V	0.1 m	
Electrostatic inchworm motor for microrobots	80 V, 8 kHz	1 mN	[112]

## Data Availability

Not applicable.

## References

[1] Bučinskas V., Subačiūtė-Žemaitienė J., Dzedzickis A., Morkvėnaitė-Vilkončienė I. (2021). Robotic micromanipulation: A) actuators and their application. Robot. Syst. Appl..

[2] Yazdani M., Payam A.F. (2015). A comparative study on material selection of microelectromechanical systems electrostatic actuators using Ashby, VIKOR and TOPSIS. Mater. Des..

[3] Baniukevic J., Hakki Boyaci I., Goktug Bozkurt A., Tamer U., Ramanavicius A., Ramanaviciene A. (2013). Magnetic gold nanoparticles in SERS-based sandwich immunoassay for antigen detection by well oriented antibodies. Biosens. Bioelectron..

[4] German N., Ramanaviciene A., Ramanavicius A. (2021). Dispersed Conducting Polymer Nanocomposites with Glucose Oxidase and Gold Nanoparticles for the Design of Enzymatic Glucose Biosensors. Polymers.

[5] Ramanavicius S., Ramanavicius A. (2021). Conducting Polymers in the Design of Biosensors and Biofuel Cells. Polymers.

[6] Ramanavicius S., Ramanavicius A. (2021). Charge Transfer and Biocompatibility Aspects in Conducting Polymer-Based Enzymatic Biosensors and Biofuel Cells. Nanomaterials.

[7] Ramanavicius S., Jagminas A., Ramanavicius A. (2021). Advances in Molecularly Imprinted Polymers Based Affinity Sensors (Review). Polymers.

[8] Wilson S.A., Jourdain R.P.J., Zhang Q., Dorey R.A., Bowen C.R., Willander M., Wahab Q.U., Willander M., Al-hilli S.M., Nur O. (2007). New materials for micro-scale sensors and actuators: An engineering review. Mater. Sci. Eng. R Rep..

[9] Esfahani S., Esmaeilzade Khadem S., Ebrahimi Mamaghani A. (2019). Size-dependent nonlinear vibration of an electrostatic nanobeam actuator considering surface effects and inter-molecular interactions. Int. J. Mech. Mater. Des..

[10] Srikar V.T., Spearing S.M. (2003). Materials selection for microfabricated electrostatic actuators. Sens. Actuators A Phys..

[11] Zhang W.M., Yan H., Peng Z.K., Meng G. (2014). Electrostatic pull-in instability in MEMS/NEMS: A review. Sens. Actuators A Phys..

[12] Veroli A., Buzzin A., Frezza F., de Cesare G., Hamidullah M., Giovine E., Verotti M., Belfiore N.P. (2018). An approach to the extreme miniaturization of rotary comb drives. Actuators.

[13] Yang Q., Xu L., Zhong W., Yan Q., Gao Y., Hong W., She Y., Yang G. (2020). Recent Advances in Motion Control of Micro/Nanomotors. Adv. Intell. Syst..

[14] Gao Y., You Z., Zhao J. (2015). Electrostatic comb-drive actuator for MEMS relays/switches with double-tilt comb fingers and tilted parallelogram beams. J. Micromech. Microeng..

[15] Li H., Ruan Y., You Z., Song Z. (2020). Design and fabrication of a novel MEMS relay with low actuation voltage. Micromachines.

[16] Ma B., You Z., Ruan Y., Chang S., Zhang G. (2016). Electrostatically actuated MEMS relay arrays for high-power applications. Microsyst. Technol..

[17] Liu C.-X., Yan Y., Wang W.-Q. (2020). Resonances and chaos of electrostatically actuated arch micro/nanoresonators with time delay velocity feedback. Chaos Solitons Fractals.

[18] Alcheikh N., Ouakad H.M., Mbarek S.B., Younis M.I. (2021). Static and dynamic actuations of clamped-clamped V-shaped micro-resonators under electrostatic forces. Mech. Syst. Signal Process..

[19] Schroedter R., Yoo H.W., Brunner D., Schitter G. (2021). Charge-Based Capacitive Self-Sensing With Continuous State Observation for Resonant Electrostatic MEMS Mirrors. J. Microelectromech. Syst..

[20] Aryal N., Emadi A. (2020). A Method to Enhance Stroke Level of a MEMS Micromirror with Repulsive Electrostatic Force. Micromachines.

[21] Xia C., Qiao D., Song X., Song X., Zheng W., He Y., Wu B. (2021). A time division capacitive feedback method of electrostatic MEMS mirror driven by PWM signal. Sens. Actuators A Phys..

[22] Varghese V., Padmanabhan R. (2020). Design and development of an electrostatic-based micropump. Int. J. Biomechatron. Biomed. Robot..

[23] Wang K.F., Wang B.L., Lin K., Li J.E., Liu Y. (2020). Nonlinear dynamics of electrostatically actuated micro-pumps with thermal effects and filled fluids. Int. J. Non-Linear Mech..

[24] Atik A.C., Özkan M.D., Özgür E., Külah H., Yıldırım E. (2020). Modeling and fabrication of electrostatically actuated diaphragms for on-chip valving of MEMS-compatible microfluidic systems. J. Micromech. Microeng..

[25] Pallay M., Miles R.N., Towfighian S. (2021). Towards a high bias voltage MEMS filter using electrostatic levitation. Mech. Syst. Signal Process..

[26] Pallay M., Towfighian S. Feasibility study of a capacitive MEMS filter using electrostatic levitation. Proceedings of the International Design Engineering Technical Conferences and Computers and Information in Engineering Conference.

[27] Hafiz M.A.A., Kosuru L., Hajjaj A.Z., Younis M.I. (2017). Highly Tunable Narrow Bandpass MEMS Filter. IEEE Trans. Electron Devices.

[28] Schaler E.W., Zohdi T.I., Fearing R.S. (2018). Thin-film repulsive-force electrostatic actuators. Sens. Actuators A Phys..

[29] Toshiyoshi H., Gianchandani Y.B., Tabata O., Zappe H. (2008). Electrostatic Actuation. Comprehensive Microsystems.

[30] Ozdogan M., Daeichin M., Ramini A., Towfighian S. (2017). Parametric resonance of a repulsive force MEMS electrostatic mirror. Sens. Actuators A Phys..

[31] Rosa M.A., De Bruyker D., Völkel A.R., Peeters E., Dunec J. (2004). A novel external electrode configuration for the electrostatic actuation of MEMS based devices. J. Micromech. Microeng..

[32] Qiao D.-Y., Yuan W.-Z., Li X.-Y. (2007). A two-beam method for extending the working range of electrostatic parallel-plate micro-actuators. J. Electrost..

[33] Chiou J.C., Lin Y.J. (2005). A novel large displacement electrostatic actuator: Pre-stress comb-drive actuator. J. Micromech. Microeng..

[34] Wang W., Wang Q., Ren H., Ma W., Qiu C., Chen Z., Fan B. (2016). Electrostatic repulsive out-of-plane actuator using conductive substrate. Sci. Rep..

[35] Zamanzadeh M., Azizi S. (2020). Static and dynamic characterization of micro-electro-mechanical system repulsive force actuators. J. Vib. Control.

[36] Schaler E.W., Jiang L., Lee C., Fearing R.S. (2018). Bidirectional, Thin-Film Repulsive-/Attractive-Force Electrostatic Actuators for a Crawling Milli-Robot. Proceedings of the MARSS 2018—International Conference on Manipulation, Automation and Robotics at Small Scales.

[37] Pallay M., Daeichin M., Towfighian S. (2017). Dynamic behavior of an electrostatic MEMS resonator with repulsive actuation. Nonlinear Dyn..

[38] Huang J., Jiang C., Li G., Lu Q., Chen H. (2021). Design and analysis of a light-operated microgripper using an opto-electrostatic repulsive combined actuator. Micromachines.

[39] Daeichin M., Ozdogan M., Towfighian S., Miles R. (2019). Dynamic response of a tunable MEMS accelerometer based on repulsive force. Sens. Actuators A Phys..

[40] Nabavi S., Menard M., Nabki F. Surface Micromachined Out-of-plane Electrostatic MEMS Actuator Integrated with Displacement Sensor. Proceedings of the IEEE Sensors.

[41] Rabenimanana T., Walter V., Kacem N., Le Moal P., Bourbon G., Lardiès J. (2021). Enhancing the linear dynamic range of a mode-localized MEMS mass sensor with repulsive electrostatic actuation. Smart Mater. Struct..

[42] Li H., Barnes P., Harding E., Duan X., Wang T.D., Oldham K.R. (2019). Large-displacement vertical electrostatic microactuator dynamics using duty-cycled softening/stiffening parametric resonance. J. Microelectromech. Syst..

[43] Abbasalipour A., Palit P., Pourkamali S. High-Energy Density Micro-Machined Cellular Arrays of Electrostatic Actuators. Proceedings of the 2019 20th International Conference on Solid-State Sensors, Actuators and Microsystems & Eurosensors XXXIII (TRANSDUCERS & EUROSENSORS XXXIII).

[44] Nabae H., Ikeda K. (2018). Effect of elastic element on self-excited electrostatic actuator. Sens. Actuators A Phys..

[45] Poletkin K. (2021). On the static pull-in of tilting actuation in electromagnetically levitating hybrid micro-actuator: Theory and experiment. Actuators.

[46] Poletkin K.V., Asadollahbaik A., Kampmann R., Korvink J.G. (2018). Levitating micro-actuators: A review. Actuators.

[47] Pallay M., Towfighian S. (2018). A reliable MEMS switch using electrostatic levitation. Appl. Phys. Lett..

[48] Pallay M., Ibrahim A.I., Miles R.N., Towfighian S. (2019). Pairing electrostatic levitation with triboelectric transduction for high-performance self-powered MEMS sensors and actuators. Appl. Phys. Lett..

[49] Pallay M., Towfighian S. A Combined MEMS Threshold Pressure Sensor and Switch. Proceedings of the 2019 IEEE Sensors.

[50] Pallay M., Miles R.N., Towfighian S. (2019). Merging parallel-plate and levitation actuators to enable linearity and tunability in electrostatic MEMS. J. Appl. Phys..

[51] Pallay M., Miles R.N., Towfighian S. (2020). A Tunable Electrostatic MEMS Pressure Switch. IEEE Trans. Ind. Electron..

[52] Mousavi M., Alzgool M., Towfighian S. (2021). Electrostatic levitation: An elegant method to control MEMS switching operation. Nonlinear Dyn..

[53] Mousavi M., Alzgool M., Towfighian S. (2021). Autonomous shock sensing using bi-stable triboelectric generators and MEMS electrostatic levitation actuators. Smart Mater. Struct..

[54] Ozdogan M., Towfighian S., Miles R.N. Fabrication and Experimental Characterization of a MEMS Microphone Using Electrostatic Levitation. Proceedings of the 2019 IEEE Sensors.

[55] Ozdogan M., Towfighian S., Miles R.N. (2020). Modeling and Characterization of a Pull-in Free MEMS Microphone. IEEE Sens. J..

[56] Hasan M.N., Pallay M., Towfighian S. Threshold Pressure Sensing Using Parametric Resonance in Electrostatic MEMS. Proceedings of the 2019 IEEE Sensors.

[57] Zamanzadeh M., Jafarsadeghi-Pournaki I., Ouakad H.M. (2020). A resonant pressure MEMS sensor based on levitation force excitation detection. Nonlinear Dyn..

[58] Poletkin K. (2020). Static Pull-in Behavior of Hybrid Levitation Micro-Actuators: Simulation, Modelling and Experimental Study. IEEE/ASME Trans. Mechatron..

[59] Mousavi M., Alzgool M., Towfighian S. (2021). A MEMS Pressure Sensor Using Electrostatic Levitation. IEEE Sens. J..

[60] Mayberry M., Ludois D.C., Severson E.L. Towards Electrostatic Levitation of Rotating Machines. Proceedings of the 2020 IEEE Energy Conversion Congress and Exposition (ECCE).

[61] Wang W., Fan D., Zhu R., Wang P., Zhao Y., Wang H. (2021). Modeling and Optimization of Electrostatic Film Actuators Based on the Method of Moments. Soft Robot..

[62] Zamanzadeh M., Jafarsadeghi Pournaki I., Azizi S. (2020). Bifurcation analysis of the levitation force MEMS actuators. Int. J. Mech. Sci..

[63] Ryalat M., Damiri H.S., ElMoaqet H., AlRabadi I. (2020). An Improved Passivity-based Control of Electrostatic MEMS Device. Micromachines.

[64] Nashat S.E.D., AbdelRassoul R., Abd El Bary A.E.M. (2018). Design and simulation of RF MEMS comb drive with ultra-low pull-in voltage and maximum displacement. Microsyst. Technol..

[65] Daeichin M., Miles R., Towfighian S. (2020). Lateral pull-in instability of electrostatic MEMS transducers employing repulsive force. Nonlinear Dyn..

[66] Burugupally S.P., Perera W.R. (2019). Dynamics of a parallel-plate electrostatic actuator in viscous dielectric media. Sens. Actuators A Phys..

[67] Nemirovsky Y., Bochobza-Degani O. (2001). A methodology and model for the pull-in parameters of electrostatic actuators. J. Microelectromech. Syst..

[68] Lee K.B. (2007). Closed-form expressions for pull-in parameters of two-degree-of-freedom torsional microactuators. J. Micromech. Microeng..

[69] Aliasghary M., Mobki H., Ouakad H.M. (2022). Pull-in Phenomenon in the Electrostatically Micro-switch Suspended between Two Conductive Plates using the Artificial Neural Network. J. Appl. Comput. Mech..

[70] Kloub H. (2020). Effect of Mechanical Loading and Increased Gap on the Dynamic Response of Multiple Degree of Freedom Electrostatic Actuator. Proceedings.

[71] Gholami R., Ansari R. (2018). Grain size and nanoscale effects on the nonlinear pull-in instability and vibrations of electrostatic actuators made of nanocrystalline material. Mater. Res. Express.

[72] Kandula P., Dong L. (2018). Robust Voltage Control for an Electrostatic Micro-Actuator. J. Dyn. Syst. Meas. Control Trans. ASME.

[73] Zhou Y., Shafai C. (2017). Reduction of Electrostatic Control Voltage with a Tri-Electrode Actuator. Proceedings.

[74] Li C., Dean R.N., Flowers G.T. (2017). Analysis and dynamic simulation of the synthetic voltage division controller for extending the parallel plate actuator stable range of motion. Microsyst. Technol..

[75] Ak C., Yildiz A., Akdagli A. (2018). A novel expression obtained by using artificial bee colony algorithm to calculate pull-in voltage of fixed-fixed micro-actuators. Microsyst. Technol..

[76] Alneamy A., Al-Ghamdi M., Park S., Khater M., Abdel-Rahman E., Heppler G. (2019). Dimpled electrostatic MEMS actuators. J. Appl. Phys..

[77] Ouakad H.M. (2017). Comprehensive numerical modeling of the nonlinear structural behavior of MEMS/NEMS electrostatic actuators under the effect of the van der Waals forces. Microsyst. Technol..

[78] Moradweysi P., Ansari R., Hosseini K., Sadeghi F. (2018). Application of modified Adomian decomposition method to pull-in instability of nano-switches using nonlocal Timoshenko beam theory. Appl. Math. Model..

[79] Hajarian A., Zand M.M., Zolfaghari N. (2019). Effect of Dispersion Forces on Dynamic Stability of Electrostatically Actuated Micro/Nano-Beams in Presence of Mechanical Shocks. Int. J. Appl. Mech..

[80] Bhojawala V.M., Vakharia D.P. (2017). Closed-form solution for static pull-in voltage of electrostatically actuated clamped—Clamped micro/nano beams under the effect of fringing field and van der Waals force Closed-form solution for static pull-in voltage of electrostatically actuated cla. Mater. Res. Express.

[81] Admassu D., Durowade T., Velicu S., Sivananthan S., Gao W. (2021). Estimation of the mechanical stiffness constant of MEMS-based parallel-plate micro-actuators. Microsyst. Technol..

[82] Sano C., Ataka M., Hashiguchi G., Toshiyoshi H. (2020). An electret-augmented low-voltage MEMS electrostatic out-of-plane actuator for acoustic transducer applications. Micromachines.

[83] Alcheikh N., Ramini A., Al Hafiz M.A., Younis M.I. (2017). Tunable clamped-guided arch resonators using electrostatically induced axial loads. Micromachines.

[84] Schmitt L., Hoffmann M. (2021). Large stepwise discrete microsystem displacements based on electrostatic bending plate actuation. Actuators.

[85] Ma W., Ma C., Wang W. (2018). Surface micromachined MEMS deformable mirror based on hexagonal parallel-plate electrostatic actuator. J. Phys. Conf. Ser..

[86] Elshenety A., El-Kholy E.E., Abdou A.F., Soliman M., Elhagry M.M. (2020). A flexible model for studying fringe field effect on parallel plate actuators. J. Electr. Syst. Inf. Technol..

[87] Ma Z., Jin X., Guo Y., Zhang T., Jin Y., Zheng X., Jin Z. (2021). Pull-In Dynamics of Two MEMS Parallel-Plate Structures for Acceleration Measurement. IEEE Sens. J..

[88] Zhou G., Dowd P. (2003). Tilted folded-beam suspension for extending the stable travel range of comb-drive actuators. J. Micromech. Microeng..

[89] Izawa T., Sasaki T., Hane K. (2017). Scanning micro-mirror with an electrostatic spring for compensation of hard-spring nonlinearity. Micromachines.

[90] Thewes A.C., Schmitt P., Löhler P., Hoffmann M. (2021). Design and characterization of an electrostatic constant-force actuator based on a non-linear spring system. Actuators.

[91] Ghalandarzadeh M., Afrang S. (2021). A new wide tunability MEMS based variable capacitor using two separate electrostatic vertical comb drive actuators. Int. J. Eng. Trans. B Appl..

[92] Velosa-Moncada L.A., Aguilera-Cortes L.A., González-Palacios M.A., Raskin J.P., Herrera-May A.L. (2018). Design of a novel MEMS microgripper with rotatory electrostatic comb-drive actuators for biomedical applications. Sensors.

[93] Zhao R., Qiao D., Song X., You Q. (2017). The exploration for an appropriate vacuum level for performance enhancement of a comb-drive microscanner. Micromachines.

[94] Akiyama T., Shono K. (1993). Controlled Stepwise Motion in Polysilicon Microstructures. J. Microelectromech. Syst..

[95] Li L., Brown J.G., Uttamchandani D. (2002). Study of scratch drive actuator force characteristics. J. Micromech. Microeng..

[96] Sarajlic E., Yamahata C., Berenschot E., Tas N., Fujita H., Krijnen G. (2010). High-performance shuffle motor fabricated by vertical trench isolation technology. Micromachines.

[97] Basset P., Kaiser A., Bigotte P., Collard D., Buchaillot L. A large stepwise motion electrostatic actuator for a wireless microrobot. Proceedings of the Fifteenth IEEE International Conference on Micro Electro Mechanical Systems.

[98] Donald B.R., Levey C.G., McGray C.G., Paprotny I., Rus D. (2006). An untethered, electrostatic, globally controllable MEMS micro-robot. J. Microelectromech. Syst..

[99] Esteves Moreira E., Lima V., Serra Alves F., Cabral J., Gaspar J., Rocha L.A. (2016). Full-gap tracking system for parallel plate electrostatic actuators using closed-loop control. Sens. Actuators A Phys..

[100] Woo J., Hahn B., Ahn C. (2020). Position estimator design for a mems top-drive electrostatic rotary actuator. Sensors.

[101] Kedzierski J., Chea H. (2021). Multilayer microhydraulic actuators with speed and force configurations. Microsyst. Nanoeng..

[102] Kurmendra, Kumar R. (2021). A review on RF micro-electro-mechanical-systems (MEMS) switch for radio frequency applications. Microsyst. Technol..

[103] Chen C., Zhang T. (2019). A review of design and fabrication of the bionic flapping wing micro air vehicles. Micromachines.

[104] Phung H., Nguyen C.T., Jung H., Nguyen T.D., Choi H.R. (2020). Bidirectional tactile display driven by electrostatic dielectric elastomer actuator. Smart Mater. Struct..

[105] Carneiro F., Zhang G., Osada M., Yoshimoto S., Yamamoto A. (2021). An Extended Model for Ripple Analysis of 2–4 Phase Resonant Electrostatic Induction Motors. Actuators.

[106] Albukhari A., Mescheder U. (2021). Investigation of the dynamics of a 2-DoF actuation unit cell for a cooperative electrostatic actuation system. Actuators.

[107] Abbasalipour A., Palit P., Sheikhlari S., Pakdelian S., Pourkamali S. (2021). High-Output Micro-Machined Electrostatic Actuators. Res. Sq..

[108] Muttikulangara S.S., Baranski M., Rehman S., Hu L., Miao J. Diffraction grating integrated on micromachined stepper motor for diversity implementation in imaging spectroscopy. Proceedings of the Fifteenth IEEE International Conference on Micro Electro Mechanical Systems.

[109] Saito K., Contreras D.S., Takeshiro Y., Okamoto Y., Hirao S., Nakata Y., Tanaka T., Kawamura S., Kaneko M., Uchikoba F. (2019). Study on Electrostatic Inchworm Motor Device for a Heterogeneous Integrated Microrobot System. Trans. Jpn. Inst. Electron. Packag..

[110] Shukla R., Beera G., Dubey A., Sharma V.P., Sankar P.R., Dhawan R., Tiwari P., Sinha A.K. (2021). Design analysis and fabrication of side-drive electrostatic micromotor by UV-SLIGA. J. Micromanuf..

[111] Jin C., Zhang J., Xu Z., Trase I., Huang S., Dong L., Liu Z., Usherwood S.E., Zhang J.X.J., Chen Z. (2020). Tunable, Flexible, and Resilient Robots Driven by an Electrostatic Actuator. Adv. Intell. Syst..

[112] Contreras D.S., Pister K.S.J. Dynamics of electrostatic inchworm motors for silicon microrobots. Proceedings of the International Conference on Manipulation, Automation and Robotics at Small Scales.

[113] Naito Y., Nakamura K., Uenishi K. (2019). Laterally movable triple electrodes actuator toward low voltage and fast response RF-MEMS switches. Sensors.

[114] Uvarov I.V., Kupriyanov A.N. (2019). Stiction-protected MEMS switch with low actuation voltage. Microsyst. Technol..

[115] Wang Q., Wang W., Zhuang X., Zhou C., Fan B. (2021). Development of an electrostatic comb-driven mems scanning mirror for two-dimensional raster scanning. Micromachines.

[116] Liu X., Takahashi T., Konishi M., Motohara K., Toshiyoshi H. (2020). Random access addressing of MEMS electrostatic shutter array for multi-object astronomical spectroscopy. Micromachines.

[117] Wang W.C., Gu K., Tsui C.L. (2019). Design and fabrication of a push-pull electrostatic actuated cantilever waveguide scanner. Micromachines.

[118] Leroy E., Hinchet R., Shea H. (2020). Multimode Hydraulically Amplified Electrostatic Actuators for Wearable Haptics. Adv. Mater..

